# Case report: Double mutations in a patient with early-onset Alzheimer’s disease in China, PSEN2 and IDE variants

**DOI:** 10.3389/fnins.2024.1423892

**Published:** 2024-10-30

**Authors:** Zhongzheng Chang, Zhiyang Wang, Lele Luo, Zhaohong Xie, Caibin Yue, Xianli Bian, Hui Yang, Ping Wang

**Affiliations:** ^1^Department of Neurology, the Second Hospital of Shandong University, Jinan, China; ^2^Cheeloo College of Medicine, Shandong University, Jinan, China; ^3^Department of Infectious Diseases and Hepatology, the Second Hospital of Shandong University, Jinan, China

**Keywords:** Alzheimer’s disease, PSEN2, IDE, EOAD, missense mutation

## Abstract

Alzheimer’s disease (AD) is a progressive neurodegenerative disease characterized by gradual cognitive decline. Early-onset Alzheimer’s disease (EOAD) is defined as AD occurring before age 65. The main pathogenic gene variants associated with EOAD include *PSEN1*, *PSEN2*, and *APP. IDE* gene has been identified as a risk factor in the pathogenesis of AD. In this study, we report a 33-year-old male with mutations in the *PSEN2* gene (c.640G > T, p.V214L) and *IDE* gene (c.782G > A, p.R261Q). *PSEN2* V214L has been reported in five previous cases, and no reported cases have carried *IDE* R261Q. He had progressive memory decline, his sister carried the same gene mutations but had no clinical manifestations. Neuroimaging revealed mild cortical atrophy. The concentration of Aβ42 in cerebrospinal fluid (CSF) was obviously decreased. In silico predictive models suggested that these mutations are damaging. Our findings indicate that mutations in the *PSEN2* and *IDE* genes may disrupt the normal functioning of their respective proteins, contributing to the pathogenesis of AD.

## 1 Introduction

Alzheimer’s disease (AD) is a neurodegenerative disease characterized by chronic progressive cognitive impairment. The typical histopathologic changes include neuroinflammatory plaques formed by excessive deposition of β-amyloid, and neurofibrillary tangles formed by aggregation of hyperphosphorylated tau protein (Tahami Monfared et al., 2022). According to whether the age of onset is less than 65 years, AD can be further categorized into early-onset AD (EOAD) and late-onset AD (LOAD) (Amaducci et al., 1986). EOAD accounts for approximately 5–10% of all cases. Compared with LOAD, EOAD usually progresses more rapidly, leading to shorter survival time and a poorer prognosis (Adriaanse et al., 2014). Presenilin-1 (*PSEN1*), Presenilin-2 (*PSEN2*), and Amyloid precursor protein (*APP*) are the main pathogenic genes associated with EOAD, among which *PSEN2* gene mutation is the rarest (Lanoiselée et al., 2017; Xiao et al., 2021). Mutations in the *PSEN2* gene enhance γ-secretase activity, leading to increased Aβ42 production. In addition, the insulin degrading enzyme (*IDE*) gene has been considered as a candidate risk factor for the pathogenesis of AD. This enzyme has the capacity to degrade β-amyloid (Ling et al., 2003). IDE deficiency reduces Aβ clearance, contributing to its Aβ deposition. During the preclinical stage, the brain has undergone pathophysiologic changes, but significant symptoms have not yet manifested (Sperling et al., 2011). The protracted preclinical stage presents a vital opportunity for early intervention, highlighting the importance of timely recognition of AD.

In this study, we report a 33-year-old man with mutations in both the *PSEN2* gene (c.640G > T, p.V214L) and the *IDE* gene (c.782G > A, p.R261Q). This report aims to enhance the understanding of the clinical phenotypic features and the spectrum of genetic mutations, we systematically detailed the clinical features of the case as well as the two gene mutations, which will help clinicians better recognize AD.

## 2 Methods

### 2.1 Clinical evaluation

The patient’s medical, personal, and family histories were obtained through interview. Also the detailed physical examination was performed on the patient. Additionally, the patient underwent a series of neuropsychological assessments, including the Mini-Mental State Examination (MMSE), Montreal Cognitive Assessment (MoCA), Clinical Dementia Rating (CDR), the 23-item Alzheimer’s Disease Cooperative Study - Activities of Daily Living (ADCS-ADL23), Hachinski Ischemic Score (HIS), Hamilton Anxiety Scale (HAMA), and Hamilton Depression Scale (HAMD). He underwent hematological examinations, including routine blood tests, erythrocyte sedimentation rate (ESR), C-reactive protein (CRP), liver and kidney function tests, blood lipid profile, blood glucose levels, myocardial enzymes panel, thyroid function tests, electrolytes, folic acid, vitamin B12, homocysteine, virus series, paraneoplastic antibodies. He also underwent brain magnetic resonance imaging (MRI). Lumbar puncture was performed for cerebrospinal fluid (CSF) analysis (routine examination, biochemical examination, Aβ40, Aβ42, total tau protein, phosphorylated tau protein, demyelinating antibodies, oligoclonal bands and paraneoplastic antibodies).

### 2.2 Genetic analysis and in silico analyses

Allele frequencies were obtained from the gnomAD database.^1^ The 168 dementia-related genes were detected by high-throughput sequencing (Supplementary material). The source of the panel was Xiangyin Medical Laboratory in China. The mutations in *PSEN2* and *IDE* were confirmed by standard Sanger sequencing. The pathogenicity of mutations was predicted using PolyPhen2 and SIFT software. CADD Scores were obtained on website,^2^ and the CADD model GRCh37-v1.7 was chosen. RCSB PDB:Homepage^3^ was used to obtain 3D protein structures of PSEN2 and IDE. The predicted structures of PSEN2 and IDE after the mutation was obtained by using Missense3D.^4^ Discovery Studio 4.5 Visualizer and Pymol were used for visualization and highlighting the mutated amino acids.

## 3 Case presentation

**FIGURE 1:**
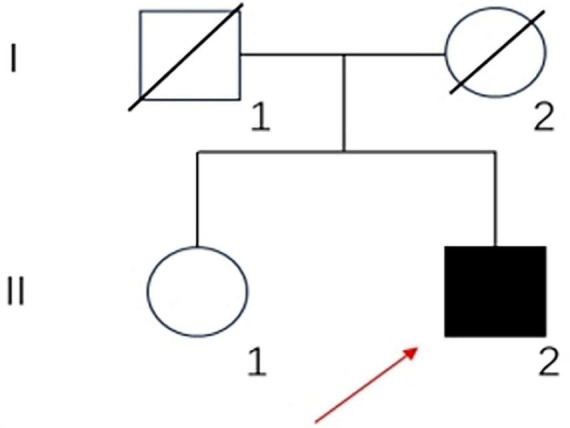
Family pedigree of the patient: square, male; circle, female; diagonal black line, deceased individual; full black filled symbol, affected individual; empty symbol, clinically healthy relative; long red arrow, proband.

A 33-year-old, primary school-graduated male presented with a 2-year history of progressive short-term memory decline. Initially, he forgot what he had just said or done. After 1 year, the condition continued to worsen. He often forgot dates and his calculation ability decreased. The cognitive decline gradually affected his daily life and work capacity. The patient has not presented hallucinations, delusions or personality changes since the onset of the disease. There were no family histories of similar diseases. His father died of a brain hemorrhage, his mother died of esophageal cancer, and his sister has no similar medical history (Figure 1).

The physical examination showed no significant abnormalities in internal medicine examination. Neurological examination showed the impairment of memory, calculation, time orientation, executive, abstract thinking and visuospatial abilities.

**FIGURE 2:**
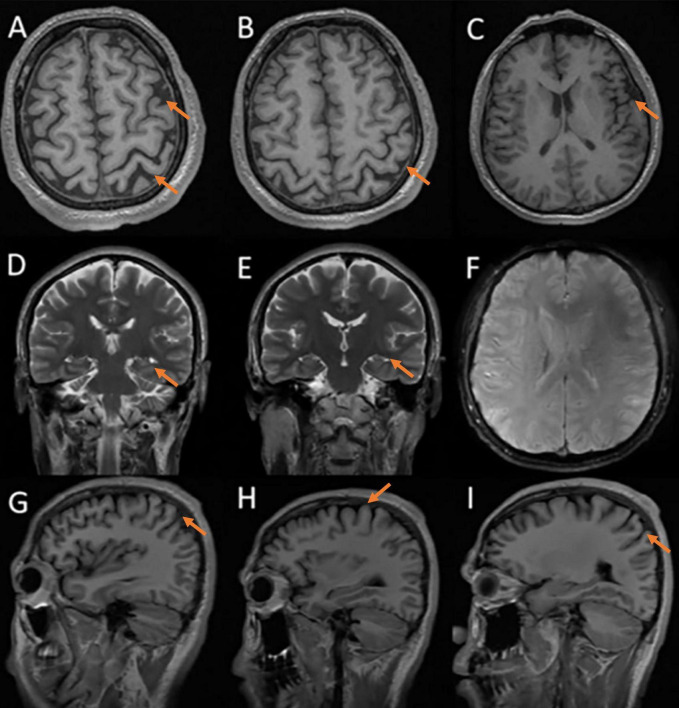
Cerebral MRI: mild cortical atrophy, SWI is normal. **(A–C)** axial T1 images; **(D,E)** coronal T2 images; **(F)** susceptibility-weighted imaging; **(G–I)** sagittal T1 images.

Neuropsychological assessments showed that his MMSE score was 15/30, MoCA score was 9/30, CDR score was 1/3, ADCS-ADL23 score was 65/80, HAMA score was 18, and HAMD score was 21, HIS score was 0. Cranial MRI in our hospital revealed mild cortical atrophy of the brain, susceptibility weighted imaging (SWI) is normal (Figure 2), Peripheral blood tests showed a slightly elevated low density lipoprotein (LDL) value of 3.59 mmol/L while parameters remained within normal ranges. The levels of the four core biomarkers of AD in CSF were as follows: Aβ42, 502.06 pg/ml (reference range ≥ 888.1 pg/ml); Aβ42/Aβ40, 0.104 (reference range ≥ 0.068); p-Tau181,15.00 pg/ml (reference range ≤ 42.0 pg/ml); T-Tau,100.00 pg/ml (reference range ≤ 378.0 pg/ml). Other CSF analyses, including CSF routine, biochemistry, demyelinating antibodies, oligoclonal bands and paraneoplastic antibodies were all normal.

**FIGURE 3:**
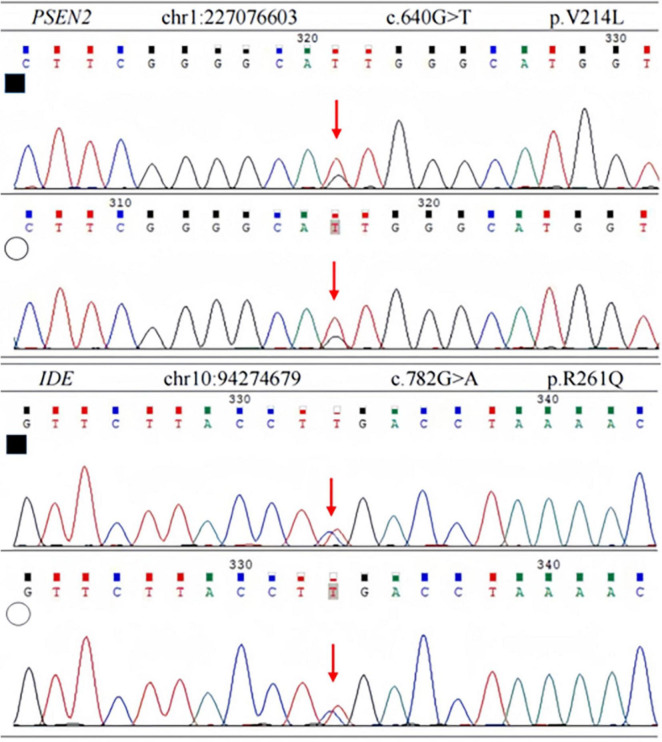
Sanger sequencing of PSEN2 and IDE variant. Each heterozygous variant is indicated by a red arrow; full black filled square, affected individual; empty circle, his sister.

**FIGURE 4:**
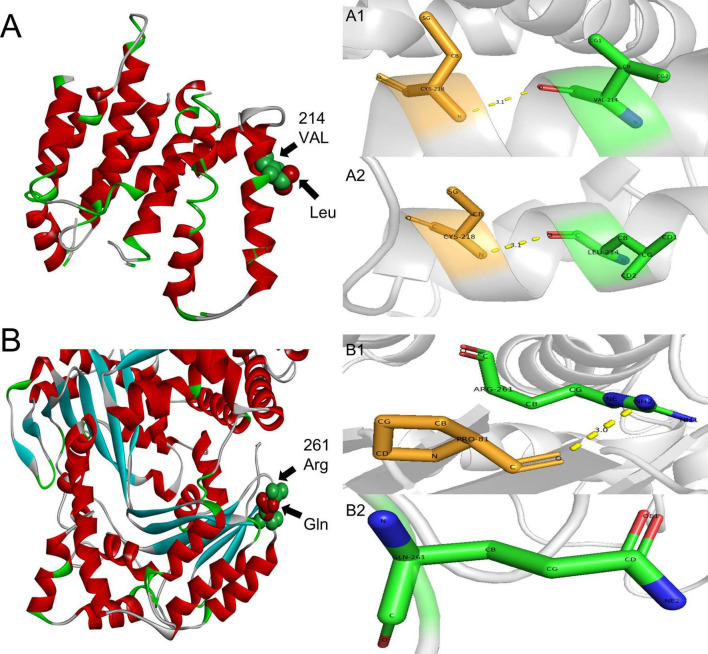
3D structure prediction of the *PSEN2* and *IDE*. **(A)** *PSEN2*, green indicates side chains of natural Val residues and red indicates Leu mutation. (A1,A2): *PSEN2*, the length of the hydrogen bond do not change after the mutation. A1: Local position of wild-type Val214. A2: Local position of mutant-type Leu214. **(B)** *IDE*, green indicates side chains of natural Arg residues and red indicates Gln mutation. (B1,B2): *IDE*: the hydrogen bond is missing after the mutation. B1: Local position of wild-type Arg261. B2: Local position of mutant-type Gln261.

In order to identify the cause of the disease, we applied high-throughput sequencing of the targeted region to detect 168 dementia-related genes and performed Sanger sequencing to detect clinically significant mutations. We found no other genetic variants in this panel, except for *PSEN2* and *IDE*. The patient’s *APOE* genotype was *APOE3 (ε3/ε3)*. His sister also carried the same mutations in *PSEN2* (c.640G > T, p.V214L) and the *IDE* (c.782G > A, p.R261Q) (Figure 3). The first mutation is in the *PSEN 2* gene: c.640G > T, p.V214L (nucleotide 640 in the coding region was mutated from guanine to thymine, resulting in a change from valine to leucine at amino acid 214), which is a missense mutation. The allele frequency in the gnomAD database is 0.000151. The SIFT prediction is “tolerable.” We used PolyPhen 2 to evaluate *PSEN2* Val214Leu mutation. The HumDiv score was 0.803 (sensitivity: 0.84; specificity: 0.93), which indicated a probably damaging mutation. The CADD score for this variant is 25.0, indicating a predicted deleterious effect on gene function. The second mutation is in the *IDE* gene: c.782G > A, p.R261Q (nucleotide 782 in the coding region mutated from guanine to adenine, resulting in a change from arginine to glutamine at amino acid 261), a missense mutation. The allele frequency in the gnomAD database is 0.00001. The SIFT prediction is “affect protein function.” Similarly, PolyPhen2 predicted that this mutation was possibly damaging, with a HumDiv score was 0.969 (sensitivity: 0.77; specificity: 0.95). The CADD score for this variant is 26.9, indicating the pathogenicity of the mutation. Upon structural prediction of native and mutant PSEN2 and IDE by using Discovery Studio and Pymol, the results revealed significant structural changes in the affected region (Figure 4).

## 4 Discussion

In this study, we report a 33-year-old male (*APOE3 ε3/ε3*) who carried mutations in both the *PSEN2* (c.640G > T, p.V214L) and *IDE* (c.782G > A, p.R261Q) genes, presenting with a 2-year history of chronic progressive memory decline. Based on the patient’s history, physical examination, laboratory results, and imaging, other possible etiologies, such as metabolic, inflammatory, tumor, infection, endocrine, toxic, traumatic or cerebrovascular etiologies were excluded. Multiple clinical assessments indicated impairments of his memory, numeracy, executive function, abstract thinking and visuospatial skill, reflecting a broad dysfunction of the cerebral cortex and related association fibers. According to the 2021 International Working Group (IWG) diagnostic criteria, the patient was diagnosed with probable AD, supported by his cerebrospinal fluid test was positive for Aβ amyloid but negative for tau protein (Dubois et al., 2021). Since the age of onset was under 65 years, the patient was diagnosed with EOAD. As AD is a persistently progressive illness, we suppose that more pathological changes involving tau proteins may occur as the disease advances. Additionally, Hendriks et al. (2024) found 15 risk factors associated with the early-onset dementia, which included low educational level. He had only 3 years of education due to poor learning ability. So low education level is a risk factor for this patient. In silico analyses strongly predicted the pathogenicity of both mutations that might affect the function of PSEN2 and IDE.

The *PSEN2* gene, localized on chromosome lq42.13, contains 12 exons. Its expression product, the PSEN2 protein, is one of the possible catalytic centers of γ-secretase complex and contains 448 amino acids and 9 transmembrane domains (Jia et al., 2020; Henricson et al., 2005). γ-secretase is a complicated tetrameric membrane-embedded protease (Yang et al., 2019), it can cleave amyloid precursor protein (APP). This cleavage process is a key step in the production of amyloid (Aβ), and abnormal aggregation of Aβ is one of the main pathological features of AD (Zheng and Koo, 2006). Abnormalities in PSEN2 protein disrupt γ-secretase activity, which leads to the excessive Aβ deposition inside and outside central nervous system cells. *PSENs/APP* missense mutations increased significantly when the proportion of EOAD in the family was >75% (Jia et al., 2020). To date, the *PSEN2* V214L has been identified in 6 cases (including the present case) in Asia (Gan et al., 2023; An et al., 2016; Shi et al., 2015; Youn et al., 2014), and has not been reported in any other region or ethnicity. 5 of these 6 cases were EOAD. The present case is the earliest in age, at 33 years. Sanger sequencing confirmed that his sister also carried this mutation at the same locus. However, she did not present AD-related clinical manifestations. There was no relevant medical history in their family, which may be related to the low penetrance of the V214L mutation in the *PSEN2* gene (Koriath et al., 2020).

This patient carried *PSEN2* gene V214L mutation, and functional predictions indicated that the mutation was pathogenic. The *PSEN2* V214L mutation is located within the transmembrane region of the PSEN2 protein (Jia et al., 2020). The non-polar amino acid residues usually constitute the hydrophobic core of the proteins, and mutations in these region are predispose to protein misfolding (De Marothy and Elofsson, 2015), resulting in protein structural changes and dysfunction. Furthermore, the highly conserved nature of this mutation site also indicates that mutation at this site are more likely to affect PSEN2 protein. In 2014, a Korean scholar used RaptorX 3D modeling to study the structural changes of presenilin 2 protein caused by mutation at this site, suggesting that this mutation would be deleterious (Youn et al., 2014). While several studies have suggested that the clinical significance of the *PSEN2* V214L mutation remains uncertain (Xiao et al., 2021), *in silico* analyses from PolyPhen-2 and CADD strongly indicated deleterious effect on gene function.

The *IDE* gene is localized on chromosome 10 q23-q25 and consists of 25 exons. The coding sequence shows highly conserved throughout evolution. As a Zn2+ metalloprotease with 1019 amino acids and a molecular weight of 110-kDa (Tian et al., 2023), *IDE* shows high evolutionary homology across species, including mice, Drosophila, bacterial and human. Many studies have found that the *IDE* expression products are distributed in nearly all organs and tissues (Authier et al., 1996), with the highest level of expression in the liver, testis, muscle, and brain. IDE consists of two domains, IDE-N and IDE-C, which are connected by a ring containing 28 amino acid residues that forms a tridentate cavity (Shen et al., 2006). Substrates and products can move freely in a “open-form” IDE, only when IDE N-terminal and C-terminal are folded to form a “closed-form,” the catalytic site can be completely formed (Manolopoulou et al., 2009). IDE can degrade amyloid-forming peptides, such as insulin, Aβ, glucagon, pancreatic amylase, cardiac natriuretic peptide, calcitonin, and insulin-like growth factors I and II. which form β-rich lamellar amyloid fibrils under specific conditions (Kurochkin, 2001). Research has shown that the activity of IDE is reduced in LOAD, but its expression level remains unchanged (Kim et al., 2007). Therefore, we hypothesize that *IDE* gene mutation alters IDE protein structure, leading to decreases enzymatic activity and diminished Aβ degradation capacity.

The *IDE* gene is thought to play a significant role in AD pathogenesis. Studies in AD animal models have shown that increased *IDE* expression correlates with cognitive improvement, decreased expression of Aβ and *APP*, and enhanced degradation of Aβ in brain tissue (Tian et al., 2023). As one of the primary enzymes responsible for degrading Aβ, *IDE* can degrade both endogenous Aβ peptides and synthetic Aβ fragments (Wang et al., 2006). Farris et al. (2004) found that rats with *IDE* missense mutations exhibited decreased catalytic efficiency and impaired Aβ degradation, suggesting that *IDE* may be a risk gene for AD. In another study, Farris et al. (2003) observed that mice with double knockout of the *IDE* gene had a significant decrease in Aβ degradation rates and elevated levels of the APP intracellular domain (AICD), which can be regarded as a pathological hallmark of AD. Moreover, *IDE* may be involved in the AD pathogenesis by influencing tau protein phosphorylation through the insulin signaling system. Hong and Lee (1997) demonstrated that glycogen synthase kinase-3β (GSK-3β) is involved in tau hyperphosphorylation, leading to the aggregation of tau proteins within neurons and the formation of neurofibrillary tangles—one of the hallmarks of AD. Insulin is also known to regulate GSK-3β activity (Lesort et al., 1999), and the activation of insulin receptors suppresses GSK-3β (Rickle et al., 2004). Some scholars have suggested that recycling of internalized insulin receptors back to the cell membrane may require separation from insulin molecules, and IDE in the early endosomes can remove insulin bound to the insulin receptor. Therefore, *IDE* deficiency may impair insulin receptor recycling (Sousa et al., 2021), leading to dysregulated insulin signaling. This disruption may enhance GSK-3β activity, leading to increased tau phosphorylation (Sousa et al., 2021). In this case, the patient carried the *IDE* R261Q mutation, which was predicted to be pathogenic. This finding, combined with current research on *IDE* function, supports the hypothesis that *IDE* gene variants may act as risk factors for EOAD (Ertekin-Taner et al., 2004).

The *APOE ε4* allele plays a vital role in facilitating Aβ production and deposition (Mahley et al., 2006). However, the patient carried the *APOE ε3/ε3* genotype, suggesting that Aβ deposition is more likely linked to the *PSEN2* V214L and *IDE* R261Q mutations. We speculate that the synergistic effect of these two genes mutations contributed to EOAD, which may also explain why the onset age of memory decline in our case was earlier. Notably, the patient’s sister, who also carries these mutations, has not presented any AD-related clinical manifestations. This suggests that pathogenic gene mutations do not always cause disease in all carriers, as AD is influenced by both genetic and modifiable risk factors (Zhang et al., 2021). *PSEN2* mutations are known to have incomplete penetrance (Xiao et al., 2021). We speculate low gene penetrance or modifiable risk factors may be involved in the development of AD. Low penetrance may be result from many factors, including genetic background, environmental exposures, and lifestyle. These factors may act individually or in combination to influence gene expression and phenotypic manifestation. Individuals with mutations in *APP* or *PSEN1* are certain to develop AD, and those with mutations in *PSEN2* have a 95% chance of developing AD (Liu et al., 2024). This study did not perform more tests, such as plasma biomarkers, AD-related Aβ-PET, Tau-PET, and functional metabolic imaging of the brain. Moreover, the patient’s parents are deceased, rendering the family tree incomplete and the source of the genetic mutations unclear. His sister did not have any memory issues, so she refused to be tested for relevant biomarkers. She was only detected mutations in *PSEN2* and *IDE*, not the full panel of targeted genes. Although some studies have shown that *PSEN2* V214L and *IDE* R261Q mutations are associated with AD pathogenesis and specific pathological changes, more reports and functional studies are necessary to definitively determine the pathogenic roles of these mutations in AD development.

## 5 Conclusion

The case involves a 33-year-old male with EOAD. He carried a rare but potentially pathogenic *PSEN2* gene mutation (c.640G > T, p.V214L), which has been reported in only 6 cases to date; He also carried *IDE* gene mutation (c.782G > A, p.R261Q), which has not previously been reported in AD patients. We reported for the first time that a patient with the EOAD carried these gene mutations. This case enriches the genetic and clinical phenotype spectrum of EOAD, providing new insights for genotyping and risk prediction. Interestingly, the patient’s sister carried the same gene mutations but has no clinical manifestations, suggesting that the development of AD results from the interplay of multiple factors. *PSEN2* V214L and *IDE* R261Q mutations might be involved in pathogenicity, since *in silico* analyses predicted the pathogenicity, although the exact mechanisms remain unclear. In the future, the functions of *PSEN*2 V214L and *IDE* R261Q mutations should be verified by cell and animal experiments to clarify their pathogenicity.

## Data Availability

The datasets presented in this study can be found in online repositories. The names of the repository/repositories and accession number(s) can be found in this article/Supplementary material.

## Footnotes

1
http://www.gnomad-sg.org/

2
https://cadd.bihealth.org/snv

3
https://www.rcsb.org/

4
http://missense3d.bc.ic.ac.uk/missense3d/
